# Revisiting Ravn virus as the lesser known orthomarburgvirus

**DOI:** 10.1038/s44298-026-00180-x

**Published:** 2026-02-17

**Authors:** Ivet A. Yordanova, Joseph B. Prescott

**Affiliations:** Centre for Biological Threats and Special Pathogens, Robert Koch Institute, Berlin, Germany

**Keywords:** Diseases, Microbiology

## Abstract

Marburg virus (MARV) is a highly pathogenic zoonotic filovirus. The *Orthomarburgvirus marburgense* species includes MARV and Ravn virus (RAVV), which differs from MARV by 21% at the nucleotide level and 22% at the protein level. This review offers fresh discussions of the epidemiology, genetics and natural reservoir transmission of RAVV, summarizes experimental animal models, outlines current vaccine development and raises outstanding questions about RAVV life history, transmission and pathogenicity.

## Introduction

The *Filoviridae* family includes nine genera—*Cuevavirus, Dianlovirus, Loebevirus Oblavirus, Striavirus, Tapjovirus, Thamnovirus, Orthoebolavirus* and *Orthomarburgvirus*. Of those, only the latter two contain viruses known to be pathogenic in humans – four belonging to the *Orthoebolavirus* genus (Ebola (EBOV), Sudan (SUDV), Taï Forest (TAFV) and Bundibugyo (BDBV) viruses) and two belonging to the *Orthomarburgvirus* genus—Marburg (MARV) and Ravn (RAVV) viruses^1^. With similar virion structures, replication strategies and pathogenicity, these orthoebolaviruses and orthomarburgviruses are etiologic agents of a group of filovirus diseases, characterized by the rapid development of severe systemic infection associated with a cytokine storm—the uncontrolled release of diverse tissue-damaging pro-inflammatory cytokines and chemokines. Other features of infection include high levels of viremia, fever, coagulation abnormalities, vascular leakage, severely deregulated innate and adaptive immune responses that ultimately result in multiorgan failure and shock, sometimes accompanied by signs of hemorrhage and a characteristic petechial rash^2–5^. In humans, median case fatality rates reach 67%, but can vary between 0% and 100%, at least in part due to differences in the numbers of confirmed cases, the country where an outbreak was reported and where confirmed cases received medical treatment.

**Fig. 1:**
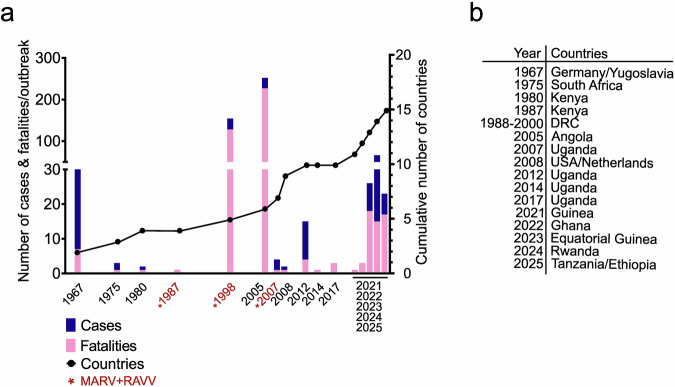
History of MVD outbreaks. **a** Graphical representation of the numbers of reported cases (blue bars) and fatalities (pink bars) of orthomarburgvirus-caused MVD outbreaks (left y-axis). The cumulative number of countries reporting orthomarburgvirus-caused outbreaks is superimposed as the black line (right y-axis). Each black dot corresponds to the cumulative number of countries reported to have had at least one orthomarburgvirus-caused outbreak, including the outbreak recorded in that year. Outbreaks with confirmed cases of RAVV are highlighted in red. **b** Table description listing the countries which have reported cases of MVD since 1967.

Public health authorities have so far recorded 19 orthomarburgvirus-associated outbreaks across 15 countries, including three outbreaks with confirmed cases of RAVV infections (Fig. 1a). Importantly, recent cases of MVD have been reported from 6 countries with no prior historical records of MVD outbreaks – an indication of the expanding frequency and geographic range of orthomarburgvirus outbreaks (Fig. 1a). The first recorded outbreak of MVD involved the simultaneous reporting of 29 cases and 7 fatalities from a novel viral disease among laboratory workers in Germany and the former Yugoslavia in 1967 leading to the discovery of MARV^6^. The most recent outbreak was reported in Ethiopia in November 2025 and included 14 confirmed cases of MARV, nine of which were fatal (Fig. 1b).

Twenty years after the first MARV outbreak, a single case of MVD in Kenya in 1987 led to the discovery of RAVV^7^. The newly-discovered virus was later designated as a distinct member of the *Orthomarburgvirus marburgense* species, expanding the number of viruses within the species to two—MARV and RAVV^7,8^. The origins of most MVD outbreaks are consistently associated with various caves or mines frequented by either miners or tourists who have come in close contact with roosting fruit bats, their saliva or excretions. Numerous experimental and ecological studies have now unequivocally confirmed that cave-dwelling Egyptian rousette bats (ERBs, *Rousettus aegyptiacus*) are natural reservoirs of both orthomarburgviruses by successfully isolating genetically diverse MARV and RAVV from wild-caught bats. They’ve also demonstrated, at least for MARV, year-round seropositivity in juvenile and adult bats, as well as detectable shedding of infectious MARV particles^9–16^. As such, ERBs represent the first and only confirmed wildlife reservoir of human-pathogenic filoviruses.

Phylogenetic analysis of MARV and RAVV classifies them into 2 distinct lineages – RAVV and MARV-Musoke, -Popp, -Angola and -Ozolin. Despite belonging to the same species and having the same natural reservoir, MARV and RAVV differ both at the nucleotide and protein level by more than 20%^9,10^. While various studies have made significant advances in our understanding of MARV pathogenicity, reservoir biology and vaccine design, there has been limited characterization of RAVV at the molecular, cellular and ecological level^17–20^. The observed genetic variation between the two pathogens could have significant influence over virus transmission dynamics, pathogenicity, host responses to treatment and vaccination. The objective of this review, therefore, is to examine and discuss the existing literature on RAVV biology and to emphasize the need for further characterization of RAVV from diverse perspectives.

## Genetics and evolution

**Fig. 2:**
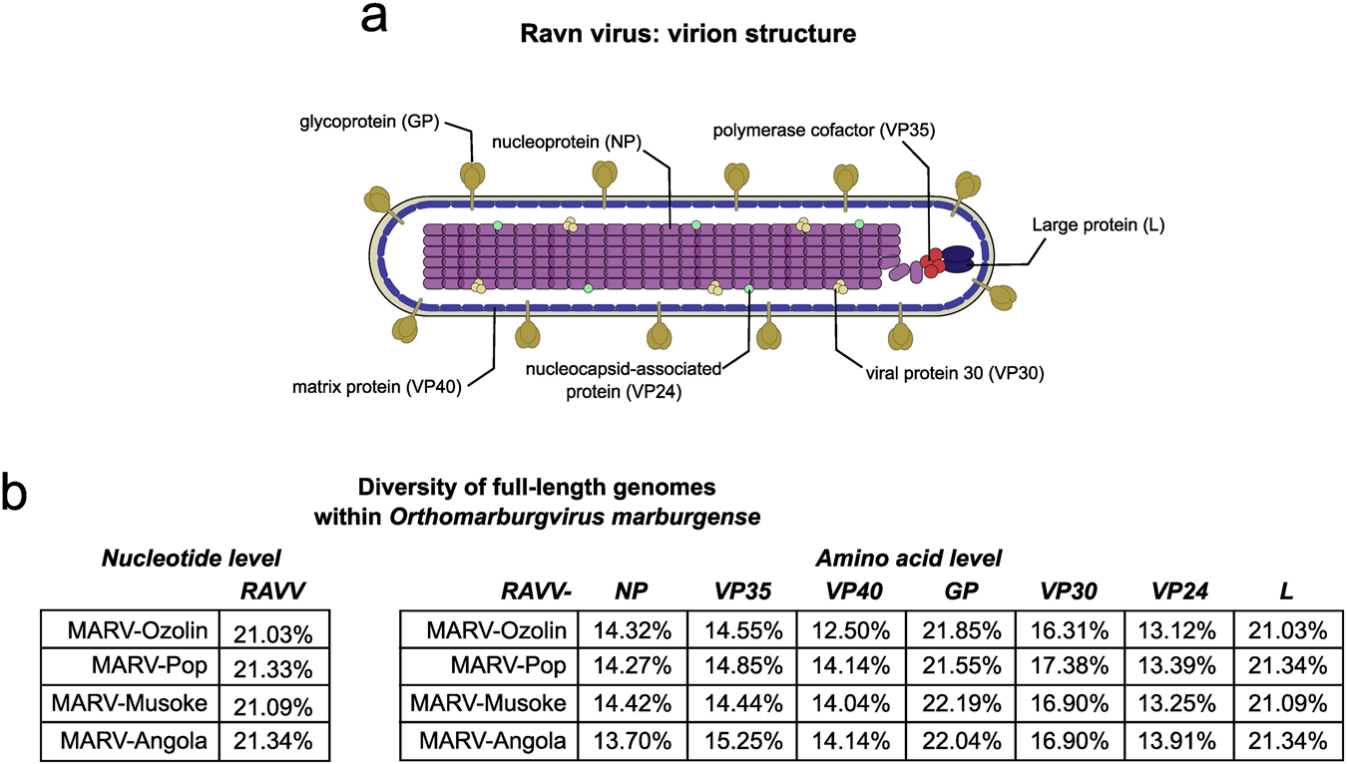
Genetics of RAVV virus. **a** Basic graphical illustration of the typical virion structure of RAVV. **b** Table summary of the percentage differences in nucleotide sequences (left table) and amino acid sequences (right table) of full-length RAVV and MARV isolates. The sequence similarity comparison in the two tables is based on the following strains: RavKen1987 (accession number DQ447649), MARV-Ozolin (accession number AY358025), MARV-Pop (accession number NC001608), MARV-Musoke (accession number DQ217792) and MARV-Angola (accession number DQ447653).

As members of the *Filoviridae* family, MARV and RAVV both possess a negative-sense, non-segmented RNA genome roughly 19 kb in length that encodes seven genes in the following order: 3’-NP-VP35-VP40-GP-VP30-VP24-L-5’ ^21^. Like MARV, the seven RAVV genes encode a nucleoprotein (NP), polymerase cofactor (VP35), matrix protein (VP40), glycoprotein (GP_1,2_), viral protein 30 (VP30), nucleocapsid-associated protein (VP24) and a Large protein (L) (Fig. 2a). Sequencing analysis of the original RAVV isolate obtained from the 1987 Kenya case (RavKen1987) has shown that its full-length genome differs from the MARV-Angola, -Musoke, -Popp and -Ozolin isolates by an average 21%, while at the amino acid level RAVV differed from these MARV isolates by up to 22%^7,9^ (Fig. 2b). Overall, the most conserved genes between RAVV and MARV include NP, VP35, VP40 and VP24 (12–15% nucleotide differences), while GP and L show the greatest nucleotide differences (21-22%)^9^ (Fig. 2b).

Examining the evolution and emergence of MARV and RAVV, current calculations estimate the molecular evolutionary rate of both MARV and RAVV at approximately 5.67 × 10^−4^ nucleotide substitutions/site/year, lower than the evolutionary rates of EBOV and the non-pathogenic Reston orthoebolavirus (RESTV), but higher than that of SUDV^22^. Finally, the most recent common ancestor (MRCA) for MARV and RAVV was estimated at around 700 years ago, meaning that the two viruses likely underwent divergent evolution in the early 14th century^22^.

In 2017, one group proposed updated sequence-based filovirus taxon demarcation criteria based on nucleotide and amino acid sequence identities following analysis of existing filovirus sequences using Pairwise Sequence Comparison (PASC). Based on their findings, they proposed an updated species demarcation criteria threshold for filoviruses in the range of 23-36%, correcting the previously established threshold of 30%^23^. As the 21% and 22% nucleotide and amino acid sequence differences between MARV and RAVV closely approach these species demarcation criteria, the potential importance of RAVV as a possible separate orthomarburgvirus species should therefore be more closely considered in any future work on MVD therapeutics and vaccine development^23^.

## Epidemiology and clinical disease

During clinical surveillance of viral hemorrhagic fever diseases in Kenya in 1987, a single fatal case of MVD led to the discovery and initial partial characterization of RAVV^7^. The patient was a 15-year-old male tourist admitted to a hospital with a history of headaches, malaise, anorexia, fever and vomiting following initial treatment for suspected malaria. Approximately nine days prior to the onset of initial symptoms, he visited Kitum Cave in Mount Elgon National Park, Kenya. The patient eventually succumbed to disease on the 11th day of illness. One viral isolate (RavKen1987) was obtained from a serum sample collected on the ninth day of illness and a cross-neutralization assay confirmed that the new isolate is closely related to MARV-Musoke, with partial sequencing of GP indicating a 72% nucleotide identity with MARV-Musoke and 71% with MARV-Popp^7^.

Between October 1998 and September 2000, a cluster of 154 cases of MVD (106 suspected and 48 confirmed) were reported in the Democratic Republic of the Congo (DRC)^24^. The mean age of the patients was 28 years, 68% of cases were male and most were miners working in Goroumbwa mine. Some of the primary cases subsequently spread the infection to family members or healthcare workers. The overall case fatality rate (CFR) during the DRC outbreak was later calculated to be 83%^24^. Nucleotide sequencing of VP35 captured at least nine distinct orthomarburgvirus lineages in circulation during this outbreak, including a new 09DRC1999 RAVV isolate detected in one patient, indicating a single spillover event of RAVV paralleled by multiple MARV spillover events in other individuals (Table 1). During the two-year span of this outbreak, a seasonal pattern of new infections was noted, transmission beginning in October/November and peaking January/February^24^. The occurrence of sporadic cases with short chains of onward transmission and the protracted nature of the outbreak suggested the presence of repeated spillover events, a hypothesis also supported by the detection of multiple genetically distinct viruses^25^. Due to the concurrent circulation of multiple isolates of MARV and RAVV, isolate-specific CFRs were impossible to calculate^2,7^. The seasonality of the cases during the 1998–2000 DRC outbreak would be later corroborated by the discovery of seasonal pulses of MARV circulation in juvenile ERBs coinciding with the typical biannual birthing seasons of this species and with periods of increased risk of virus spillover to humans^11^.

**Table 1:** Nomenclature, geographic origin, host origin and sequence accession numbers of current RAVV virus isolates

Isolate abbreviation	Year and place of discovery	Host origin	GenBank Accession number
RavKen1987	1987 (Kenya)	Human	DQ447649
09DRC1999	1999 (Democratic Republic of the Congo)	Human	DQ447652
02Uga2007	2007 (Uganda)	Human	FJ750953
44Bat2007	2007 (Uganda)	Egyptian rousette bat	FJ750954
188Bat2007	2007 (Uganda)	Egyptian rousette bat	FJ750955
982Bat2007	2007 (Uganda)	Egyptian rousette bat	FJ750956
1304QBatUga2009	2009 (Uganda)	Egyptian rousette bat	JX458857
RSA-2017-bat8095	2017 (South Africa)	Egyptian rousette bat	MT321489

**Fig. 3:**
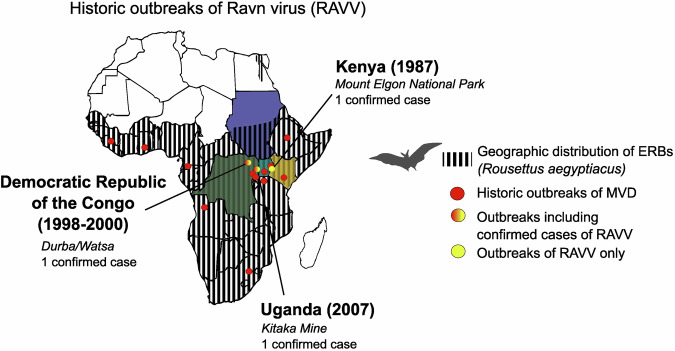
Geographic distribution of historic RAVV outbreaks. Map of the African continent with country borders, highlighting the geographic distribution of ERBs (*Rousettus aegyptiacus*, grey stripes), historic MVD outbreaks involving MARV-only (red dots), MARV + RAVV (red-yellow) or RAVV-only cases (yellow).

In 2007, following a small outbreak of MVD among members of a mining community at Kitaka mine in western Uganda, orthomarburgviruses were isolated and sequenced from two confirmed cases and the full-length genomes of two isolates were obtained—01Uga2007 and 02Uga2007^10^. Bayesian analysis revealed that the 02Uga2007 isolate clearly belongs to the RAVV lineage and is closely related to the RavKen1987 and 09DRC1999 lineages (Table 1), thus confirming the third MVD outbreak to date involving spillover of RAVV^10^ (Fig. 3).

## Natural reservoirs and transmission patterns

Among the initial indications that fruit bats are natural reservoirs of orthomarburgviruses was the fact that the patient representing the first confirmed case of RAVV infection in 1987 had visited Kitum cave in Kenya prior to the onset of symptoms^7^ (Fig. 3). More than half of the MVD cases during the 1998–2000 outbreak in DRC were associated with proximity to local gold mines that housed large multi-species bat colonies, while the single RAVV case detected in Kenya in 2007 was similarly associated with Kitaka mine (Fig. 3)^24–26^. A subsequent ecological survey across Gabon and DRC detected MARV-specific RNA and IgG antibodies in Egyptian rousette bats (ERBs, *Rousettus aegyptiacus*). Out of 285 captured ERBs, tissues obtained from 4 bats tested positive for MARV RNA via quantitative real-time PCR and 29 bats were seropositive for MARV-specific antibodies, evidencing the circulation of MARV in this species^27^. A similar ecological survey in Goroumbwa mine in DRC sampled various insectivorous and fruit bats, rodents, shrews and arthropods. Nested RT-PCR detected MARV-VP35 sequences in 8 microbats and four ERBs, while MARV IgG ELISA confirmed a 9.7% seropositivity among eloquent horseshoe bats (*Rhinolophus eloquens*) and 20.5% seropositivity among ERBs^25^. Phylogenetic analysis detected the presence of 15 distinct MARV sequences in circulation during the DRC outbreak (6 in bats and 9 in humans), including several bat-derived VP35 sequences divergent by up to 21%. Given that MARV-Ozolin, - Pop, - Musoke and -Angola VP35 nucleotide sequences only differ from each other between 0.4 and 5.7% as opposed to their differences with RAVV of 14.5-15.3%, as well as the average 21% difference in whole genome sequences between RAVV and MARV isolates, it is therefore likely that the sequences with the highest level of divergence found in this study belonged to RAVV^9^.

Follow-up investigations in response to the 2007 Uganda outbreak of MVD sampled more than 1000 ERBs and various roundleaf bats (genus *Hipposideros*) and found the presence of genetically diverse orthomarburgviruses in ERBs. Among these, 31/611 bats (5.1%) tested positive for MARV RNA and 13/546 bats (2.4%) were seropositive for MARV-specific IgG antibodies, two of which were also weakly positive via qRT-PCR^10^. In addition to detecting a distinct RAVV lineage (02Uga2007) in one of the two confirmed human cases of MVD in the 2007 Uganda outbreak, phylogenetic analysis revealed that three of the bat-derived viral isolates resided within the RavKen1987 lineage and closely matched the human 02Uga2007 isolate by 99.2–99.9%, supporting the reservoir competence of ERBs for RAVV due to the presence of identical or near-identical bat-derived and human-derived RAVV isolates^10^ (Table 1). Another survey of ERBs in South Africa during peak virus transmission season detected several viral sequences in rectal samples, which shared a common ancestor with the human RavKen1987, human 1999 DRC and bat 2007 Uganda isolates, further highlighting the natural circulation and shedding of RAVV in ERBs across geographically distinct areas^14^.

Further fieldwork has shown that at peak times, 2–3% of ERBs in a given roost are actively infected with orthomarburgviruses, highlighting year-round virus maintenance in the reservoir population. Infection rates have also been associated with biannual seasonal spikes, related to this species’ birthing seasons and are linked to over 80% of documented MARV spillover events in humans. Combined with the fact that most confirmed MVD outbreaks have occurred within the geographic distribution of ERBs on the African continent further strengthens the importance of these bats as natural reservoirs of orthomarburgviruses^11^ (Fig. 3).

ERBs experimentally infected with MARV typically develop mild, transient subclinical disease accompanied by viremia which peaks around day 7, oral and rectal shedding which peak around days 9 and 7, respectively^15^. MARV infection in these bats progresses in the presence of discrete foci of inflammation in the liver and no notable histopathological lesions in other tissues, paralleled by transient seroconversion. MARV-specific IgG antibody levels peak between 12- and 28-days post-infection, followed by a rapid decline over the following three months. Despite waning humoral immunity, long-lived protection against reinfection is evident in ERBs, indicating the development and maintenance of robust immunological memory to MARV^12,15,28,29^.

Unlike MARV, the progression of RAVV infection in ERBs has so far scarcely been addressed. However, one recent study used captive-bred bats and the 188bat2007 RAVV isolate to characterize viral infection and shedding dynamics in RAVV-infected ERBs^30^. Viremia was detected in all infected bats, peaking around 5 days post-infection and with an average length of oral shedding of 8.8 days, highly comparable to MARV-infected bats. The absence of clinical signs of disease in RAVV-infected animals was also consistent with past MARV experimental studies. In line with previous work on MARV, all RAVV-infected bats demonstrated a robust humoral immune response and seroconverted by 21 days post-infection in the absence of signs of clinical disease, providing the first direct evidence that ERBs sustain RAVV replication and shedding, and mount an immune response to the virus^30^. Interestingly, RAVV-infected ERBs had an overall higher and more prolonged rectal and oral viral shedding compared with MARV-infected animals. Together with evidence from another ecological survey detecting only RAVV-positive rectal swab samples in wild-caught bats, this indicates measurable differences in viral shedding between RAVV and MARV^14,30^. These findings therefore indicate potential divergence in the ecology of MARV and RAVV transmission and in bat immune responses against the two viruses.

In addition to MARV and RAVV, ERBs are also a natural reservoir for Kasokero virus (KASV), a human-pathogenic orthonairovirus maintained in enzootic transmission cycles between ERBs and argasid ticks (*Ornithodoros faini*)^31–33^. Recent investigations showed that coinfections with MARV, KASV and Sosuga virus (SOSV), a paramyxovirus ERBs also serve as a putative natural reservoir for, can modulate viral shedding and antibody production dynamics. Importantly, recent follow-up RAVV work further evaluated whether ERBs with prior MARV or MARV + KASV co-infection experience differences in controlling subsequent MARV or RAVV challenge infections^30,34^. Consistent with previous work that describes the development of long-term protective immunity against MARV, neither the MARV-challenged nor RAVV-challenged animals in this study developed detectable viremia or any clinical signs of disease over the course of a 10-day infection. Moreover, the presence of a prior KASV infection appeared to have no influence on MARV or RAVV infection, indicating that these bats develop comparable protective immunity to experimental challenge with MARV and RAVV^34^.

## Animals models of disease

**Fig. 4:**
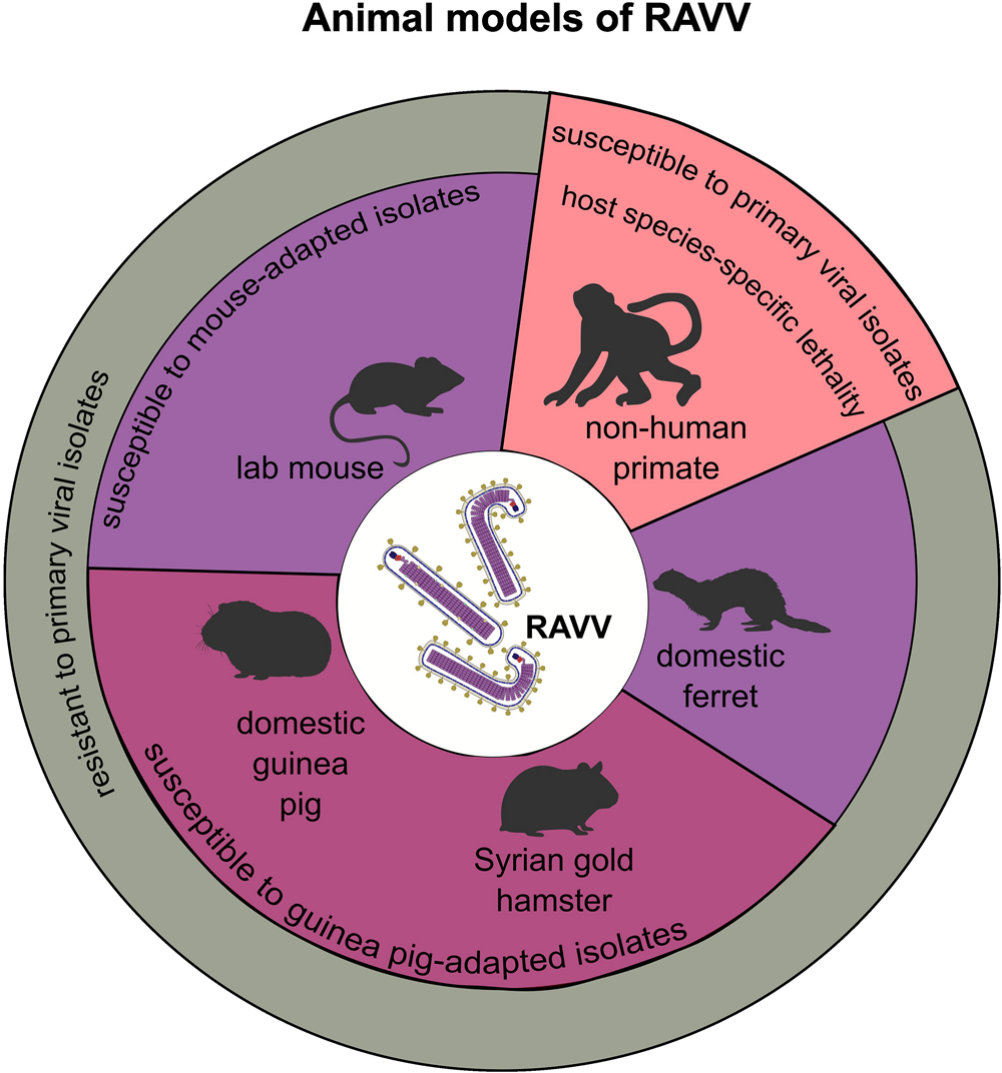
Animal models of RAVV. Graphical summary of some of the key features of currently published animal models of Ravn virus-caused MVD.

Despite belonging to the same species, the significant degree of variation between RAVV and MARV at the genome and amino acid level could contribute to potential divergence in virulence and disease outcomes between the two viruses. However, given that only 3 confirmed human cases of RAVV have been reported to date, drawing conclusions about differing disease outcomes between MARV and RAVV in humans is currently not possible. Classic experimental approaches to address differences in virulence include in vivo infections using established animal models of MVD^35,36^. However, most animal models of MVD, including various mouse and guinea pig models, have notable drawbacks due to the need to use rodent-adapted viral strains and the natural resistance of rodents to MVD caused by primary viral isolates **(**Fig. 4**)**. The rodent-adapted viral strains do not genetically match clinically relevant isolates and don’t always cause observable signs of disease that recapitulate the hallmarks of human disease^36^.

Despite some of the obvious drawbacks of lab mouse models, several studies have successfully recapitulated the main features of either orthoebolavirus or orthomarburgvirus-caused disease in humanized mice – animals devoid of their native immune responses and artificially recapitulated with a human innate and adaptive immune system. In one study, humanized bone marrow/liver/thymus (hu-BLT) mice challenged with wild-type clinical isolates of either EBOV-Mayinga or EBOV-Makona led to lethal EVD closely recapitulating the clinical, pathological and immunological findings typical of human patients with severe EVD^37^. Follow-up investigations comparing the pathogenicity of non-adapted EBOV-Makona and MARV-Angola demonstrated differential disease severity caused by each virus in triple knockout BLT (TKO-BLT) mice—a finding not typically observed in humans where EBOV and MARV are equivalently pathogenic^38^. Overall, humanized mouse models offer notable advantages over wild-type mouse strains for more accurate interrogations of specific human innate and adaptive immune cell responses to various orthoebolaviruses and orthomarburgviruses^38–40^. However, comparative studies of MARV and RAVV replication or pathogenicity using humanized mice have not been published to date. Various studies over the years have also tried but failed to recapitulate orthomarburgvirus-caused MVD in ferrets, leaving NHPs as the most reliable and clinically relevant animal model of disease caused by orthomarburgviruses (Fig. 4).

### Laboratory mice

Human isolates of MARV, such as Angola, Musoke and Ci67, as well as RAVV, are generally non-pathogenic in immunocompetent adult laboratory mice (*Mus musculus*). Serial passaging of orthoebolaviruses in suckling mice typically results in uniformly lethal infections of adult mice with the newly mouse-adapted strains^41^. Similarly, serial passaging of orthomarburgviruses in severe combined immunodeficient (SCID) mice leads to the generation of mouse-adapted strains capable of inducing some clinical signs of disease in experimentally infected mice^42,43^. However, only limited work with mouse-adapted RAVV has been reported to date.

Following initial inoculation of SCID mice with wild-type MARV-Ci67, MARV-Musoke and RAVV, mouse-adapted isolates of each virus can be generated by repeatedly passaging these isolates in SCID mice (43). Within 4 days of infection, naïve SCID adult mice infected with each adapted virus were reported to develop weight loss, a hunched posture, elevated liver enzymes, hepatocellular necrosis and viremia levels up to 10^6^ pfu/mL by day 8 post-infection^42^. Compared with MARV-Ci67 and -Musoke, RAVV adapted faster to SCID mice, evidenced by its higher lethality measured as mean time-to-death (MTD) within the first two blind passages. A follow-up study demonstrated that further passaging of SCID-adapted RAVV in immunocompetent adult BALB/c mice eventually generated a lethal mouse-adapted RAVV that caused high viremia, weight loss, severe hepatic lesions and necrosis in the spleen and liver upon challenge infection in BALB/c mice^43^. Comparison of the nucleotide sequences of non-adapted and mouse-adapted RAVV isolates revealed 61 nucleotide differences in the latter, the majority of which were located in the NP, VP35, VP40 and VP30 coding regions, highlighting that host adaptation of RAVV towards higher lethality potentially involves the accumulation of mutations in viral proteins associated with host IFN antagonism (VP35 and VP40) and genome replication (NP and VP30)^43^.

Host resistance during the initial stages of filovirus infections typically relies on the secretion of type I IFNs released from diverse host immune cell types. Using immunocompetent BALB/c and 129 mice, as well as SCID and IFN receptor (IFNAR) knockout mice, another study showed that infection with guinea pig-adapted RAVV resulted in high viral replication rates and high lethality in these immunocompromised mice. However, immunocompetent wild-type mice with intact IFN signaling maintained 100% survival following initial infection with guinea pig-adapted RAVV, indicating that an intact immune response is central to the ability of mice to control the virus^44^.

### Domestic guinea pigs

Domestic guinea pig (*Cavia porcellus*) models have previously been used to develop uniformly lethal strains of MARV by serial adaptation. Temporal comparison of the lethality of MARV-Angola and RAVV has demonstrated limited recapitulation of MVD as observed in humans and NHPs and limited strain-specific differences when using guinea pig-adapted strains of each virus^45^. MARV and RAVV-infected animals displayed comparable peaks in viremia on days 3 and 7 post-infection, with minor differences in mean viral titers in various tissues. MARV-infected animals met euthanasia criteria on average 1 day earlier than the RAVV-infected group despite comparable progression of weight loss^45^. Both viruses induced splenic enlargement and mottling, multifocal to diffuse hepatic discoloration, swollen lymph nodes and gastrointestinal ulceration, the latter two appearing more prominent in MARV-infected than RAVV-infected animals. Both adapted viruses also induced sustained neutrophilia, thrombocytopenia, increased platelet volume and infected liver-resident macrophages (Kupffer cells) beginning on day 3 of infection, indicative of comparable host cell tropism^45^. Overall, this guinea pig model was able to mimic to some degree classic MVD clinical features in humans and NHPs, including lymphopenia, thrombocytopenia, splenic and hepatic tissue damage among others^36^. Virus adaptation via serial passages led to the accumulation of several genetic mutations in RAVV-NP, -GP and -VP40 proteins. The adapted MARV acquired a single amino acid change in VP40 and two in VP24, highlighting the need to further study the importance of shared versus disparate mutations in the various viral proteins as potential molecular landmarks responsible for the differential virulence of MARV and RAVV^45^.

### Syrian gold hamsters

Comparative studies of MARV and RAVV using Syrian gold hamsters remain limited to date. Lethal hamster-adapted strains of MARV-Angola, MARV-Ci67 and RAVV that recapitulate most clinical features of MVD have previously been generated following up to five passages of guinea pig-adapted strains of each virus in hamsters^46,47^. An immunocompromised STAT2 knockout hamster strain also appears susceptible to infection with wild-type MARV-Angola, MARV-Musoke and RAVV, even though RAVV-infected animals showed delayed weight loss and 100% recovery rates by 21 days post-infection^48^. However, a more detailed interrogation of viral replication and clinical pathology in RAVV-infected hamsters is still lacking and merits further investigation.

### Domestic ferrets

Domestic ferrets (family: Mustelidae) have long been established as reliable small animal models of diverse zoonotic pathogens. Their susceptibility to human and avian influenza A viruses, SARS-CoV-2 and pneumoviruses, alongside their ability to recapitulate clinical signs of disease following infections with henipaviruses and rubulaviruses has made ferrets widely used models of zoonotic virus transmission, pathogenesis and immune responses^49–52^. Ferrets have also been tested as suitable animal models of filovirus disease caused by various orthoebolaviruses. Several studies could show that ferrets infected with wild-type viral isolates of orthoebolaviruses developed clinical signs of disease, indicating their potential suitability as small animal models of MVD as well^53–55^.

Comparative studies of MARV-Angola, MARV-Musoke and RAVV challenge infections have previously reported that orthomarburgvirus-infected ferrets develop no notable signs of weight loss, fever or viremia^53,56^. Moreover, there were no alterations in hematological or biochemical parameters following infection with either virus^53,56^. Using recombinant VSV-MARV, one study could show that ferret-derived spleen cells are permissible to MARV-GP mediated entry but produced no infectious viral particles following infection with wild-type MARV. In contrast, a significant increase in infectious viral particles was measured in ferret-derived lung cells, indicating host cell-specific differences in viral replication and a limited ability of wild-type MARV to infect ferret cells in vitro^57^. Guinea pig-adapted MARV-Angola (GPA-MARV) was recently shown to be uniformly lethal in experimentally infected ferrets by 10 days post-infection, in contrast with only partial lethality of mouse-adapted MARV (MA-MARV) in the same model^58^. Notably, animals infected with GPA-MARV developed severe weight loss, fever, inappetence, petechial rashes and several exhibited signs of hemorrhage evidenced by the detection of blood in stool samples, while MA-MARV-infected ferrets developed milder weight loss without fever or other signs of disease^58^. GPA-MARV also replicated to higher levels than MA-MARV and led to markedly elevated pro-inflammatory cytokines including IL-6, IL-8, CXCL10 and MCP-1 – classic elements of filovirus-induced cytokine storm. More importantly, sequencing revealed that GPA-MARV acquired two non-synonymous mutations in VP40 and VP24, representing reversion towards wild-type MARV-Angola, indicating a crucial role of these mutations for viral pathogenesis in vivo^58^. What roles the acquisition of various mutations potentially play in host-specific RAVV pathogenesis, however, remains to be addressed.

### Non-human primates

Various non-human primate (NHP) species have been used as models of filovirus disease caused by orthoebolaviruses and orthomarburgviruses, including common marmosets (*Callithrix jachus*), rhesus macaques (*Macaca mulatta*), cynomolgus macaques (*Macaca fascicularis*) and grivets (*Chlorocebus aethiops*), commonly known as African green monkeys and the original source of the 1967 MARV outbreak in Germany and Yugoslavia^44,59–62^. Rhesus and cynomolgus macaques generally recapitulate most accurately the typical disease features in humans and remain the most widely used experimental primate models for characterizing viral hemorrhagic fever disease progression, testing treatments and vaccines^63–67^. However, only a limited set of studies to date have leveraged the use of experimental NHP models to study RAVV pathogenesis.

Comparative analysis of the disease progression of RAVV, MARV-Musoke, -Angola and -Ozolin in rhesus and cynomolgus macaques demonstrated that MARV-Angola was lethal in both species, while MARV-Ozolin was not lethal in either species^68^. In contrast, MARV-Musoke infection caused a delayed disease progression in rhesus macaques and partial lethality in cynomolgus macaques, while RAVV was lethal in cynomolgus (comparable to MARV-Angola) but not in rhesus macaques (comparable to MARV-Ozolin)—evidence of clear host-specific pathogenicity of RAVV even in closely-related species^68^. Disease outcome in this study was proportional to viral titers in blood, with only one of two RAVV-infected rhesus macaques showing low levels of detectable viremia, while both RAVV-infected cynomolgus macaques showed rapidly increasing viremia between 3 and 9 dpi. Importantly, all animals from both primate species seroconverted in response to RAVV infection, but only cynomolgus macaques presented with elevated liver enzymes, creatinine and histological signs of acute hepatocellular and follicular necrosis^68^. Together, these results corroborate earlier findings where even though rhesus macaques inoculated with the RavKen1987 isolate showed clear and sudden onset of disease between 4–7 days post-inoculation, characterized by pronounced fever, anorexia, petechial rash and hemorrhagic diathesis, RAVV was not uniformly lethal in this species, with one of three sick animals improving and making a full recovery paralleled by seroconversion^7^. Histopathological examination of the other two monkeys showed hemorrhage in all major organs and electron microscopy revealed the presence of viral proteins in liver, lung, spleen, lymph nodes and kidneys, indicating wide-spread dissemination of RAVV in these animals^68^.

Another study used rhesus macaques to test the efficacy of monoclonal antibody (mAb)-driven protection against MARV and RAVV challenge using GP-specific mAbs derived from a human MVD survivor^45,69^. Groups of rhesus macaques were inoculated with either MARV-Angola or RAVV and received intravenous treatment with the mAb MR191-N at 5 and 8 dpi, a human GP-specific neutralizing mAb isolated from a MARV survivor^70^. In the infected groups, 80% of MARV-infected and 100% of RAVV-infected animals survived. None of the mAb-treated individuals were viremic by 11 dpi and exhibited only limited immunopathology, highlighting comparable responses to mAb treatment^69^. Moreover, these findings highlighted that the MR191 epitope recognized by the MR191-N mAb is conserved between MARV and RAVV and may be crucial for the fitness of both viruses^69^.

## Vaccine development efforts

Considering the high genetic variation between MARV and RAVV, divergent host protection induced by vaccines and therapeutics is a major potential concern. Orthomarburgvirus vaccine development has so far largely focused on targeting MARV isolates and few studies have incorporated RAVV in their vaccine design protocols^18,59,71–73^. However, several recent examples exist of experimental vaccine formulations that elicit varying degrees of cross-protection against MARV and RAVV—an important feature for the potential development of a future pan-orthomarburgvirus vaccine.

One group developed an adenovirus-vectored vaccine expressing a MARV-GP fusion protein derived from wild-type MARV-Musoke and MARV-Ci67. Immunization of mice and guinea pigs led to the generation of strong antibody responses and induced protection against both homologous (against MARV-Musoke and MARV-Ci67 following immunization with the MARV-GP expressing adenovirus vector) and heterologous (against RAVV following immunization with the MARV-GP expressing adenovirus vector) challenge—evidence of the potential for the development of a trivalent MARV vaccine with cross-protective efficacy^74^. These findings corroborated earlier work in the same group, which incorporated MARV-Musoke-, MARV-Ci67- or RAVV-GP into adenovirus-vectored vaccine formulations, demonstrating the induction of measurable homologous and heterologous humoral and cellular immune responses against the three viral GP proteins *i*n vitro, even though the study did not test protective immunity following in vivo challenge infections^75^.

Subsequent work used a virus-like particle (VLP) vaccine construct based on MARV-Musoke to investigate protection against adapted MARV-Musoke, MARV-Ci67 and RAVV in guinea pigs [61]. Overall, MARV-Musoke VLPs induced strong humoral immune responses in immunized guinea pigs, including cross-reactive antibodies against RAVV, and provided measurable cross-protection against lethal challenge with guinea pig-adapted MARV-Ci67 and RAVV. In contrast, control animals developed typical signs of clinical disease such as lethargy, weight loss, ocular bleeding and mild-to-moderate hepatitis, hepatocellular necrosis and lymphocytosis^76^. Importantly, the same vaccine formulation was then tested in cynomolgus macaques challenged with MARV-Musoke, -Ci67 and RAVV. Vaccinated macaques challenged with RAVV showed no clinical signs of disease and survived until the endpoint of the study (day 28 post-infection) with a single exception. One vaccinated animal developed mild signs of disease without detectable viremia, underlining the overall development of strong cross-protection against RAVV in both guinea pigs and NHPs with prior immunization using the MARV-Musoke-based VLP vaccine^76^.

Messenger RNA (mRNA) lipid nanoparticle-based vaccine formulations against MARV-Angola and RAVV based on the glycoprotein of each virus have also recently been developed^77^. Initial vaccination trials were conducted in guinea pigs via intramuscular administration of the vaccine and a booster shot on day 27. A single dose of each vaccine induced measurable virus-specific IgG responses by 27 days post-infection, which were further elevated following a booster dose, and both vaccines induced comparable neutralizing Abs^77^. Assessment of cross-reactive neutralizing Abs showed that the MARV vaccine induced high cross-neutralizing Ab titers against RAVV, while the RAVV vaccine induced almost 10-fold lower neutralizing Ab titers against MARV. Overall, the RAVV vaccine yielded higher RAVV-neutralizing Ab titers compared to MARV-neutralizing antibody titers generated by the MARV vaccine. MARV-specific serum antibodies had a lower binding capacity for cleaved GP than RAVV-specific antibodies, in addition to displaying differences in neutralizing specificity against different regions of the viral GP protein, indicating both qualitative and quantitative differences in vaccine-induced humoral responses generated against MARV and RAVV GP. Nevertheless, both vaccines provided a degree of cross-protection against heterologous challenge with guinea pig-adapted MARV-Angola and RAVV. MARV-vaccinated guinea pigs showed no signs of clinical disease or mortality following RAVV challenge and vice versa,(78) further emphasizing the feasibility of developing pan-orthomarburgvirus vaccines^77^.

## Concluding remarks and future perspectives

Despite RAVV and the various MARV isolates belonging to the same species, growing evidence suggests these orthomarburgviruses display not only genetic differences at the nucleotide and amino acid level, but also exhibit differential pathogenicity in primate spillover hosts and differences in oral and rectal shedding patterns in their natural bat reservoirs. Various ecological investigations have found that the entire known genetic spectrum of MARV and RAVV co-circulates in ERBs in various locations and emerging studies are only now starting to compare the replication and shedding dynamics of MARV and RAVV in these bats^10,25,30^. However, according to historical data, MARV spillovers into humans appear much more frequently than RAVV. Considering the co-circulation of the two viruses in ERBs and the higher shedding of RAVV compared with MARV observed in experimentally-infected bats, this discrepancy in spillover frequency between the two pathogens points to potentially key differences in virus ecology we have yet to understand, such as whether the significantly lower rates of spillover of RAVV compared with MARV are potentially associated with differences in viral shedding between ERB subspecies. Alternatively, whether RAVV replication and shedding in ERBs is more sensitive than MARV to reproductive or nutritional stress factors across the species’ extensive geographical distribution, reminiscent of older findings of ecological stress factors influencing the risk of Hendra virus spillover from flying foxes in Australia, should be studied^78^.

Other key questions about RAVV dynamics in the wild also remain to be addressed, such as whether RAVV undergoes horizontal transmission between infected and naïve ERBs at similar rates to MARV or whether female ERBs are capable of transmitting RAVV vertically to offspring—a mechanism recently demonstrated in the suspected EBOV reservoirs, Angolan free-tailed bats, when experimentally challenged with EBOV^79^. Additional questions include whether ERBs infected with MARV are subsequently protected from infection with RAVV and vice versa or if MARV and RAVV can co-infect individual bats, does that introduce the risk of combined spillover from the same colony. And since replication and rectal shedding appear different in MARV and RAVV-infected ERBs, are these bats potentially employing distinct strategies to control each virus? Moreover, given that ERBs are also natural reservoirs of the paramyxovirus Sosuga (SOSV) and the orthonairovirus Kasokero (KASV), the potential outcomes of a RAVV-SOSV or RAVV-KASV co-infection merit further investigation, in line with recent studies showing diminished IgG responses and MARV shedding in MARV-SOSV co-infected bats contrasted by stronger IgG responses and significantly elevated MARV shedding in the MARV-KASV co-infected group^33^.

Viral glycoproteins play a critical role in virus infectivity, stability and immune evasion and are key targets of therapeutics and vaccines. Given the known differences in RAVV and MARV GP at the amino acid level, it is therefore imperative to also address whether structural divergence in GP between MARV isolates and RAVV potentially affects virus stability, replication and shedding in their natural bat reservoirs. Whether these differences also exert potential effects on host cell infection rates and differential pathogenicity in various spillover hosts also merits further investigations. Similarly, why domestic ferrets are susceptible to wild-type orthoebolaviruses, but are refractive to MARV and RAVV merits further studies. One possibility herein could be host-specific differences in humoral immune responses. mAbs directed against the unique MARV-GP_2_ wing region (a portion of the mucin-like domain attached to GP_2_ instead of GP_1_ and not found in orthoebolaviruses) were shown to be highly-protective in mouse models^80^. Domestic ferrets could be mounting specific Ab isotype responses that efficiently neutralize orthomarburgviruses but not orthoebolaviruses, contributing to their differential susceptibility. Whether RAVV and MARV, with their 22% divergence in glycoprotein amino acid sequences, harbor differences in their GP_2_ wing regions that potentially contribute to host-specific differences in susceptibility to infection or to vaccine-induced humoral immunity should thus be addressed in future studies.

The recently reported differences in vaccine-derived neutralizing and non-neutralizing Ab responses to MARV and RAVV could potentially also influence downstream host antibody-dependent cellular functions. Moreover, the presence of cross-protection against genetically diverse orthomarburgviruses appears to depend on the vaccine platform, as a Venezuelan equine encephalitis virus replication-competent vaccine construct against MARV-Musoke reportedly fails to induce cross-protection against RAVV^77,81^. We therefore strongly emphasize on the necessity to also consider these differences in host responses to MARV and RAVV in future vaccine development efforts against MARV isolates alone, as well as in studies developing cross-protective orthomarburgvirus vaccines.

## Data Availability

No new datasets were generated or analysed during the current study.
